# A rare case of malignancy of giant cell tumor of distal end radius: a clinical image

**DOI:** 10.11604/pamj.2023.44.116.38365

**Published:** 2023-03-02

**Authors:** Yukti Jobanputtra, Deepali Patil

**Affiliations:** 1 Department of Physiotherapy, Ravi Nair Physiotherapy College, Datta Meghe Institute of Medical Sciences Sawangi, Wardha, Maharashtra, India,; 2 Department of Musculoskeletal Physiotherapy, Ravi Nair Physiotherapy College, Datta Meghe Institute of Medical Sciences Sawangi, Wardha, Maharashtra, India

**Keywords:** Giant cell tumor, X-ray, physiotherapy

## Image in medicine

A 56-year-old male patient reported to the orthopaedic department with complaints of swelling and pain in the wrist associated with discharge that makes it difficult for him to perform activities. On examination, he gave a history of aggressive growth of swelling on the wrist for 6 months along with weight loss. On inspection, it revealed a single diffuse swelling over the right wrist of size 46x47 cm circumferentially, extending from mid-forearm to the distal end of the forearm that was globular in shape with ill-defined margins proximally and an ulcer of size 6x4 cm over the swelling. The discharge was purulent, yellow-colored with a foul smell. Over the swelling tortuous veins were visible. On palpation, firm and cystic areas with increased local temperature and enlarged right, axillary lymph nodes were noted. He had an addiction to tobacco chewing for the past 12 years. Various investigations were performed: fine needle aspiration cytology, magnetic resonance imaging, X-ray of the wrist that suggested a giant cell tumor (A,B). After clinico-radiological and histopathological findings, it was found that it was a malignant type tumor hence limb salvage surgery could not be considered. Therefore, surgery was planned and stitches are shown (C) for below elbow amputation as shown in the X-ray (D). Post-surgery revealed no complications and was further referred to the physiotherapy department. The primary goal of physiotherapy was to prevent secondary complications, improve and maintain the ranges and strength of the shoulder and elbow along with improvising his quality of life.

**Figure 1:**
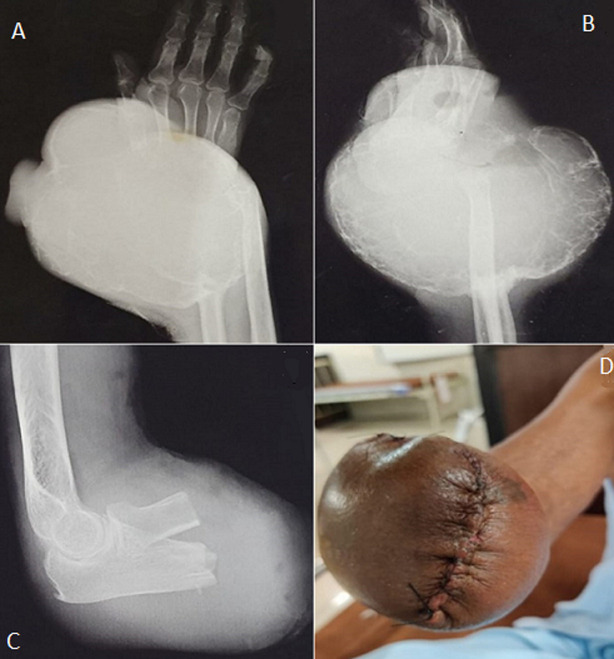
X-ray of right wrist showing giant cell tumor: A) anteroposterior view, B) lateral view; C) post-operative X-ray of elbow amputation; D) amputated limb with sutures

